# Magnetic memory driven by topological insulators

**DOI:** 10.1038/s41467-021-26478-3

**Published:** 2021-10-29

**Authors:** Hao Wu, Aitian Chen, Peng Zhang, Haoran He, John Nance, Chenyang Guo, Julian Sasaki, Takanori Shirokura, Pham Nam Hai, Bin Fang, Seyed Armin Razavi, Kin Wong, Yan Wen, Yinchang Ma, Guoqiang Yu, Gregory P. Carman, Xiufeng Han, Xixiang Zhang, Kang L. Wang

**Affiliations:** 1 Department of Electrical and Computer Engineering, and Department of Physics and Astronomy, University of California, Los Angeles, CA 90095 USA; 2 Physical Science and Engineering Division, King Abdullah University of Science and Technology, Thuwal, 23955-6900 Saudi Arabia; 3 Department of Mechanical and Aerospace Engineering, University of California, Los Angeles, CA 90095 USA; 4 Beijing National Laboratory for Condensed Matter Physics, Institute of Physics, Chinese Academy of Sciences, Beijing, 100190 China; 5 Department of Electrical and Electronic Engineering, Tokyo Institute of Technology, Tokyo, 152-8550 Japan; 6 Center for Spintronics Research Network (CSRN), The University of Tokyo, Tokyo, 113-8656 Japan

**Keywords:** Topological insulators, Electronic devices, Magnetic devices, Electronic and spintronic devices

## Abstract

Giant spin-orbit torque (SOT) from topological insulators (TIs) provides an energy efficient writing method for magnetic memory, which, however, is still premature for practical applications due to the challenge of the integration with magnetic tunnel junctions (MTJs). Here, we demonstrate a functional TI-MTJ device that could become the core element of the future energy-efficient spintronic devices, such as SOT-based magnetic random-access memory (SOT-MRAM). The state-of-the-art tunneling magnetoresistance (TMR) ratio of 102% and the ultralow switching current density of 1.2 × 10^5^ A cm^−2^ have been simultaneously achieved in the TI-MTJ device at room temperature, laying down the foundation for TI-driven SOT-MRAM. The charge-spin conversion efficiency *θ*_SH_ in TIs is quantified by both the SOT-induced shift of the magnetic switching field (*θ*_SH_ = 1.59) and the SOT-induced ferromagnetic resonance (ST-FMR) (*θ*_SH_ = 1.02), which is one order of magnitude larger than that in conventional heavy metals. These results inspire a revolution of SOT-MRAM from classical to quantum materials, with great potential to further reduce the energy consumption.

## Abstract (web summary)

It remains challenging to integrate topological insulators (TI) with magnetic tunnel junctions (MTJ) for spintronics applications. Here, the authors achieve a large tunneling magnetoresistance ratio and a low switching current density in a TI-MTJ device at room temperature, very promising for TI-driven magnetic memory.

## Introduction

Non-volatile magnetic memory is a promising candidate for next-generation memory technology beyond complementary metal–oxide–semiconductor (CMOS). Such magnetic random-access memory (MRAM)^1,2^, has ultralow energy consumption (~fJ), ultrafast speed (~ns) and almost infinite endurance (10^15^ cycles). The information of MRAM is stored in the magnetic tunnel junction (MTJ) of a ferromagnetic electrode/insulator/ferromagnetic electrode (FM/I/FM) structure, where the tunneling resistance strongly depends on the magnetization orientations of two FM electrodes, and thus the information of “0” and “1” can be stored in the parallel and antiparallel magnetization states, respectively^3–8^. Besides the magnetic field, current-induced spin torques such as spin-transfer torque (STT) and spin-orbit torque (SOT) can be used to provide an efficient switching mechanism of the magnetization^9–12^.

In particular, SOT-driven magnetization switching has been demonstrated in heavy metal/ferromagnet (HM/FM) based structures, where the spin current generated by the strong spin-orbit coupling in HMs exerts a spin torque to the adjacent FM and thus switches the magnetization with a current (typically with a density around 10^7^ A cm^−2^)^11–15^. Compared to the 2-terminal STT-MRAM in which the writing current flows vertically through the MTJ stack, in the 3-terminal SOT-MRAM, the writing current only flows transversely in a separate bottom electrode, and thus the electromigration resulting damage to the tunneling barrier can be minimized, which dramatically increases the endurance of MRAM^16–21^.

Reducing the energy consumption is a major challenge for SOT-MRAM. Quantum materials such as topological insulators (TIs) inspire a promising route to overcome the limitation of charge-spin conversion efficiency *θ*_SH_ = *J*_s_ /*J*_e_ < 1 in classical materials^22–34^, where *J*_s_ and *J*_e_ represent the spin current density and charge current density, respectively. In TIs, the topological surface states give rise to a large *θ*_SH_ due to the spin-momentum locking of the surface Dirac electrons, where the bulk is insulating in the ideal case^25,27,35^. A great interest has been focused on the SOT in TI/FM bilayer structures, in which *θ*_SH_ is found to be 1-2 orders of magnitude larger than that in HMs even at room temperature^28–33^. However, there is still a big challenge for integrating TIs with MTJs for SOT-MRAM applications: conventional SOT-MRAM is based on the current Si-based CMOS technology, whereas the single-crystal TI layer needs to be epitaxially grown on specific substrates (such as GaAs and Al_2_O_3_) by molecular beam epitaxy (MBE)^36^. Therefore, for TI-driven SOT-MRAM, the following issues need to be solved: How to control the interface between TIs and MTJs to achieve state-of-the-art tunneling magnetoresistance (TMR) ratio and at the same time preserve the topological surface states? How to reduce the element diffusion that damages the TI surface states during the annealing treatment for MTJ? How to avoid the chemical degradation of the TI crystal quality during the photolithography process of SOT devices?

In this article, we demonstrate a TI-driven SOT-MRAM cell with a state-of-the-art *TMR* ratio (over 100%) and an ultralow switching current density *J*_c_ (10^5^ A cm^−2^) at room temperature, where the topological surface states contribute to the large charge-spin conversion (*θ*_SH_ > 1). Two types of SOT switching are demonstrated: the collinear spin polarization ***σ*** and easy axis (EA), for which can realize the field-free switching with lower *J*_c_ (1.2 × 10^5^ A cm^−2^); the orthogonal ***σ*** and EA, which leads to a much faster switching speed. The charge-spin conversion efficiency *θ*_SH_ in TIs is quantified by two methods: the SOT-induced shift of the magnetic switching field and the SOT-induced ferromagnetic resonance (ST-FMR), which give rise to *θ*_SH_ = 1.59 and *θ*_SH_ = 1.02, respectively. At last, SOT switching is demonstrated in the all-sputtered TI-MTJ device for potential industry-level applications. This work demonstrates the SOT-MRAM cell driven by TIs with ultralow energy consumption.

## Results and discussion

### Device structure and magnetic properties

The full TI-MTJ stack consists of a sequence of layers: (BiSb)_2_Te_3_(10)/Ru(5)/CoFeB(2.5)/MgO(1.9)/CoFeB(5)/Ta(8)/Ru(7) (thickness in nanometers). The TI of (BiSb)_2_Te_3_ is epitaxially grown on the Al_2_O_3_(0001) substrate by the MBE method, where the layer-by-layer growth mode is monitored by the reflection high-energy electron diffraction (RHEED) patterns. Then, the (BiSb)_2_Te_3_ sample is transferred from the MBE chamber to the magnetron sputtering chamber to grow the MTJ stack of Ru(5)/CoFeB(2.5)/MgO(1.9)/CoFeB(5)/Ta(8)/Ru(7). The Ru interlayer is inserted to decouple the exchange interaction between (BiSb)_2_Te_3_ and CoFeB and block the element diffusion during annealing, which may destroy the topological surface states^37,38^. The TI-MTJ stack is patterned into the SOT devices by the photo and electron-beam lithography combined with the ion milling process. A 300 ^o^C annealing process is performed to improve the crystal quality of the MgO barrier and the *TMR* ratio, during which an in-plane magnetic field (8 kOe) is applied to induce an in-plane magnetic easy axis (EA) for the top and bottom CoFeB (T-CoFeB and B-CoFeB) electrodes^39,40^.

**Fig. 1:**
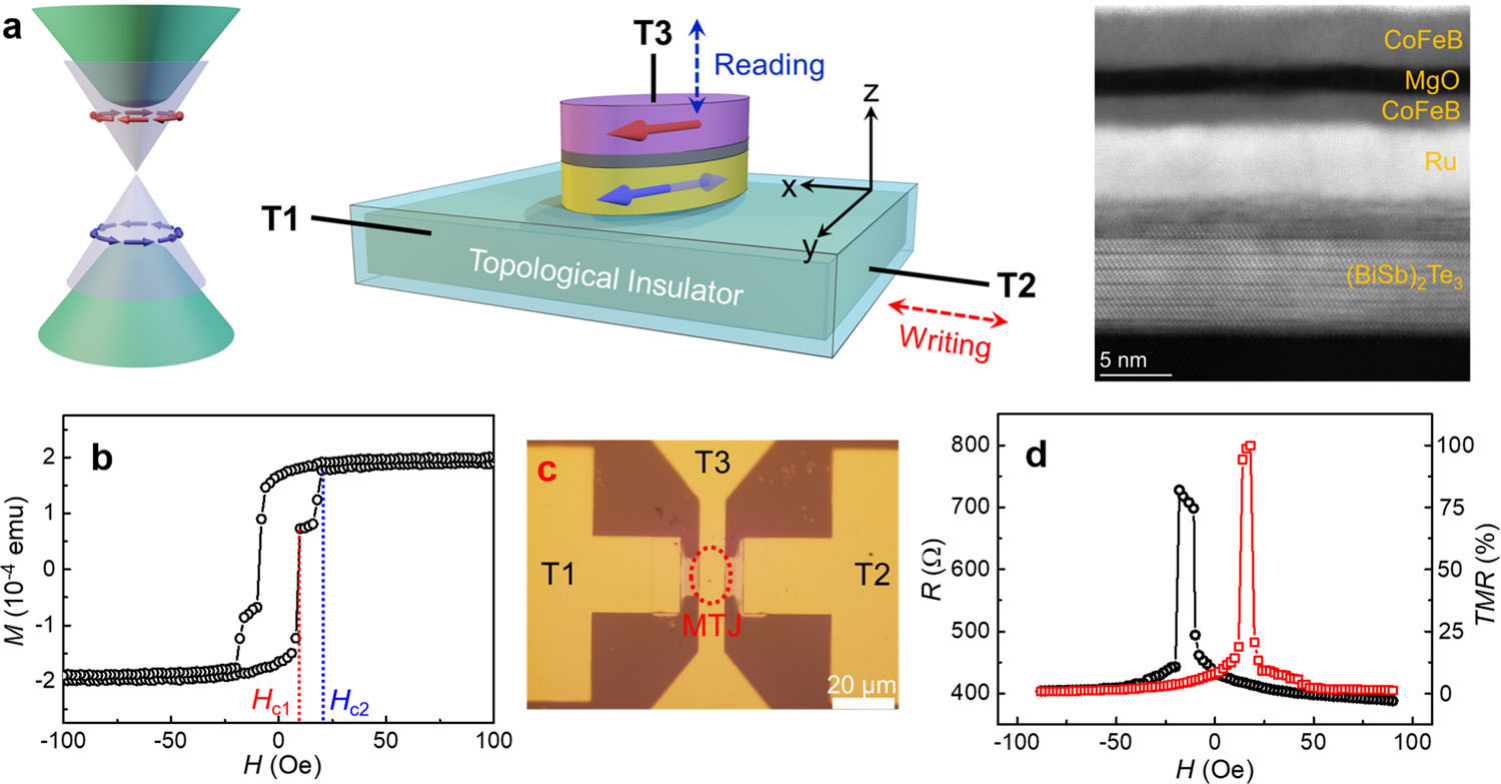
Schematic of the TI-driven SOT-MRAM cell. **a** Schematic of the 3-terminal SOT-MRAM cell with a topological insulator (TI). The writing current applied between T1 and T2 is used to switch the magnetic tunnel junction (MTJ) between the parallel and antiparallel states by the spin-orbit torque (SOT), and the reading is done by the tunneling magnetoresistance (TMR) between T1 and T3. In the TI, the surface is conducting while the bulk is insulating, and the spin-momentum locking of the surface states provides a giant SOT. Cross-sectional scanning transmission electron microscopy (STEM) results show the layer-by-layer structure of the TI [(BiSb)_2_Te_3_] and the clear interface between TI and MTJ. **b** The two-step switching process in the *M*-*H* curves shows the different coercive fields of the bottom and top CoFeB layers in the MTJ stack. **c** Microscopic picture of the patterned SOT-MRAM device, where the TI layer serves as the bottom electrode, and the MTJ device on top is 2 μm × 6 μm in size. The scale bar is 20 μm. **d** Tunneling resistance *R* and *TMR* ratio as a function of the magnetic field, where the 102% *TMR* ratio indicates the high quality of MTJ.

Figure 1a shows the helical Dirac-cone band structure of the topological surface states, where the spin-momentum locking gives rise the spin-polarized electron current. In the schematic of the 3-terminal SOT device of TI-MTJ, as shown in Fig. 1a, the writing current is applied between terminal 1 (T1) and T2, where the spin-polarized current in topological surface states is employed to provide the SOT and switch the magnetization of the free-layer (B-CoFeB) of the MTJ. For reading, a small vertical current between T1 and T3 is applied to pass through the tunneling barrier MgO, where the tunneling resistance strongly depends on the magnetization orientation between the free-layer (B-CoFeB) and the fixed-layer (T-CoFeB): low resistance for the parallel state (“0” state) and high resistance for the antiparallel state (“1” state), respectively, i.e., the TMR effect. From the cross-sectional scanning transmission electron microscopy (STEM) results, we see the layer-by-layer (i.e., van der Waals) structure of the TI [(BiSb)_2_Te_3_] and the clear interface between TI and MTJ.

The magnetic hysteresis (*M*-*H*) loop of the TI-MTJ stack is shown in Fig. 1b, where the magnetic field is scanned along the EA of the MTJ. The 2-step magnetization reversal process clearly shows the different coercive fields of T-CoFeB (*H*_c1_ = 10 Oe) and B-CoFeB (*H*_c2_ = 20 Oe), which supports an antiparallel state between *H*_c1_ and *H*_c2_. The microscopic picture of the patterned SOT device is shown in Fig. 1c, where the TI layer serves as the bottom electrode, and the MTJ stack of a 2 μm × 6 μm size is located at the intersection region between the bottom and top electrodes. Figure 1d shows the tunneling resistance *R* and the *TMR* ratio as a function of the magnetic field *H* at room temperature, where a *TMR* ratio of 102% indicates the high-quality of the MTJ on top of the TI surface.

### Current-driven SOT switching

The current-driven SOT switching is performed in two types of configurations^41^: the collinear case between ***σ*** and EA (EA along *y*) and the orthogonal case (EA along *x*). A writing current pulse *J*_e_ (1 ms) between T1 and T2 is applied to provide the SOT, followed by a small reading current pulse *J*_R_ (10 μA, 1 ms) that passes through the MTJ between T1 and T3 to detect the magnetization at 1-s later.

**Fig. 2:**
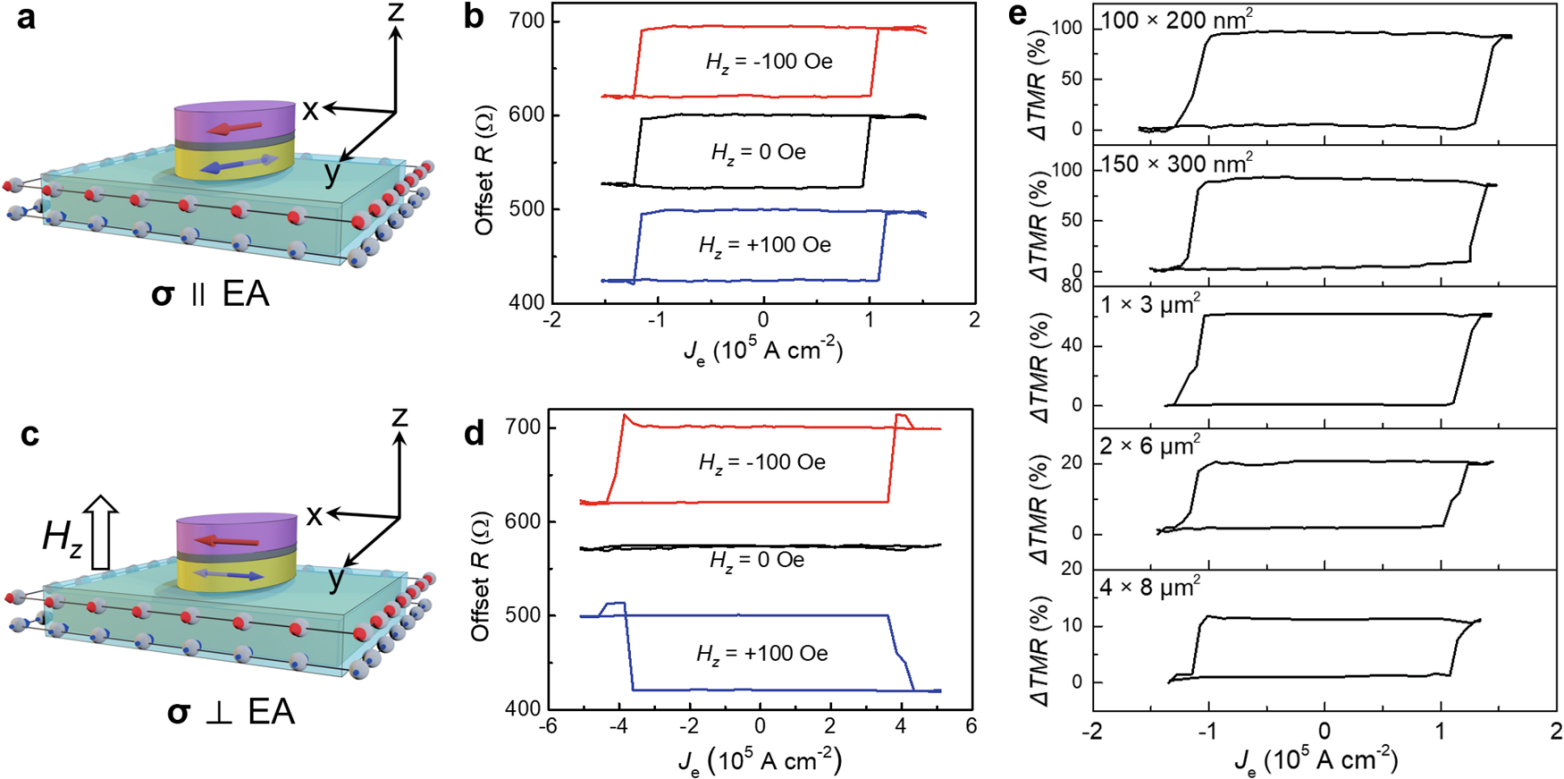
Current-driven SOT switching. **a**, **b** show the SOT switching for the collinear case between the spin polarization (***σ***) and the easy axis (***σ***∥EA). **b** The current-driven SOT switching shows the deterministic switching without external magnetic field, i.e., field-free switching, where the magnetic field *H*_*z*_ has not effect on the switching polarity. **c**, **d** show the SOT switching for the orthogonal case of ***σ***⊥EA, where a *H*_z_ is needed for deterministic switching. **d** For the ***σ***⊥EA case, an external magnetic field *H*_*z*_ is needed to break the mirror symmetry between +*m*_*x*_ and −*m*_*x*_ for deterministic SOT switching, as indicated by the opposite SOT switching polarities under *H*_*z*_ = ±100 Oe. **e**, SOT switching for the MTJ scaling down from 4 µm × 8 µm to 100 nm × 200 nm (***σ***∥EA).

For the collinear ***σ***∥EA case (EA along *y*), as shown in Fig. 2a, the damping-like torque [$-m\times (m\times \sigma )$] breaks the mirror symmetry between +*m*_*y*_ and −*m*_*y*_ and thus efficiently switches the magnetization without the external magnetic field, i.e., field-free switching. Macrospin simulation results show the magnetization gradually precesses from +*m*_*y*_ to −*m*_*y*_ states, i.e., precessional switching mode, with a switching speed of 7.5 ns (Supplementary Note 1 and Supplementary Fig. 1b). Figure 2b shows the MTJ resistance as a function of the writing current density in the TI layer (*R*-*J*_e_) at room temperature, with a critical switching current density *J*_c_ of 1.2 × 10^5^ A cm^−2^, which is 1-2 orders of magnitude lower than that in HM-based systems^11–15^.

For the orthogonal ***σ***⊥EA case (EA along *x*), an external magnetic field *H*_*z*_ is needed to break the mirror symmetry between +*m*_*x*_ and −*m*_*x*_ for the deterministic switching^41^, as shown in Fig. 2c. For ***σ***⊥EA, due to the polarity of the magnetization changes once the SOT is applied, the dynamic reversal mode produces a much shorter switching trajectory and a much faster switching speed of 1.0 ns (Supplementary Note 1 and Supplementary Fig. 1e). Figure 2d shows that *J*_c_ in the ***σ***⊥EA case (4.1 × 10^5^ A cm^−2^) is more than 3 times higher than that in the ***σ***∥EA case (1.2 × 10^5^ A cm^−2^). The opposite switching polarities at *H*_*z*_ = ±100 Oe indicate the standard SOT switching characteristic.

Only the partial SOT switching is achieved in large-size (µm) MTJs, because of the magnetic domain pining from the steps at the (BiSb)_2_Te_3_ surface induced by the layer-by-layer growth. In order to realize the full SOT switching, the size of MTJ is scaling down from 4 µm × 8 µm to 100 nm × 200 nm, as shown in Fig. 2e, and the results show that the SOT switching ratio increases with scaling down the MTJ, where the almost full SOT switching is achieved in 100 nm × 200 nm (switching ratio *ΔTMR/TMR* = 95%) and 150 nm × 300 nm (switching ratio 92%) MTJs. The MTJ size for full SOT switching is consistent with the crystal grain size (200~300 nm) of the (BiSb)_2_Te_3_ surface^42^.

### SOT-induced shift of the magnetic switching field

**Fig. 3:**
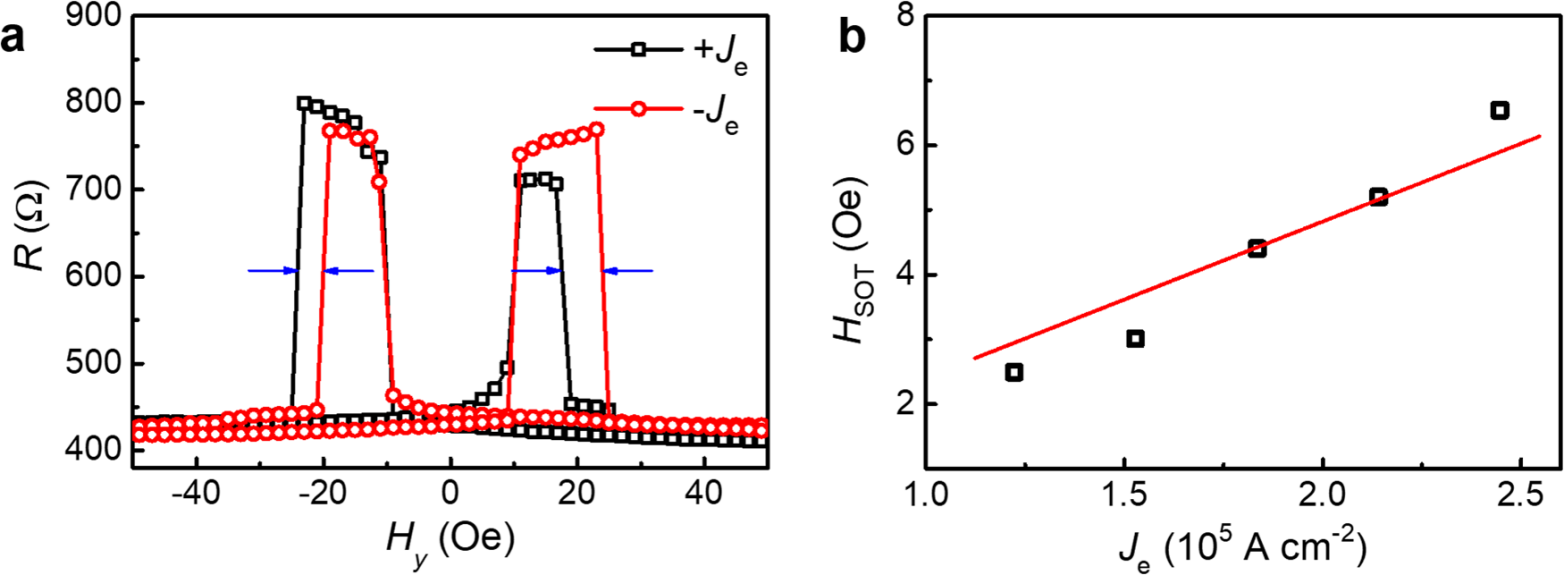
SOT-induced shift of the magnetic switching field. **a** *R*-*H*_*y*_ curves measured under the opposite bias SOT current ±*J*_e_ between T1 and T2, where the shift of the magnetic switching field of B-CoFeB indicates the SOT-induced effective field *H*_c2_^shift^ = (*H*_c2+_^shift^ + *H*_c2−_^shift^)/2 = *H*_SOT_(+*J*_e_) − *H*_SOT_(−*J*_e_) = 2*H*_SOT_(*J*_e_). **b** *H*_SOT_ as a function of the bias SOT current *J*_e_, where the linear dependence shows the typical SOT characteristic.

The SOT-induced effective field *H*_SOT_ is quantified by the shift of the magnetic switching field *H*_c2_ of B-CoFeB in the *R*-*H*_*y*_ loops^43^, under a bias SOT current *J*_e_ in the bottom electrode between T1 and T2, where EA is along the *y* axis, as shown in Fig. 3a. Due to the *H*_SOT_, the *H*_c2_ is shifted to the opposite direction under ±*J*_e_, respectively, and the shift field *H*_c2_^shift^ = (*H*_c2+_^shift^ + *H*_c2−_^shift^)/2 = *H*_SOT_( + *J*_e_) − *H*_SOT_( − *J*_e_) = 2 *H*_SOT_(*J*_e_), where *H*_c2+_^shift^ and *H*_c2−_^shift^ represent the shift of the positive and negative switching fields of B-CoFeB, respectively.

The *H*_SOT_ as a function of *J*_e_ is plotted in Fig. 3b, and the linear dependence is consistent with the spin-momentum locking induced spin polarization in topological surface states. The SOT efficiency *χ*_SOT_ = *H*_SOT_/*J*_e_ = 24.1 × 10^−6^ Oe A^−1^ cm^2^ is obtained by fitting the *H*_SOT_-*J*_e_ curve, and thus contributes to a charge-spin conversion efficiency of ${\theta }_{SH}=(2|e|{M}_{s}{t}_{F}/\hslash )\times {\chi }_{SOT}=1.59$, where *e* is the electron charge, *M*_s_ is the saturation magnetization (1100 emu cm^−3^), *t*_F_ is the magnetic film thickness (2.5 nm), and $\hslash$ is the reduced Planck constant. The obtained value of *θ*_SH_ (1.59) here is consistent with that (*θ*_SH_ = 2.5) in (BiSb)_2_Te_3_/Ti/CoFeB/MgO system with perpendicular magnetic anisotropy by the 2^nd^ harmonic measurement^31^. By considering the 2-dimentional (2D) current distribution in the TI surface, we can also obtain the interfacial charge-spin conversion efficiency ${q}_{{{{{{\rm{ICS}}}}}}}={J}_{{{{{{\rm{s}}}}}}}/{J}_{{{{{{\rm{e}}}}}}}^{2{{{{{\rm{D}}}}}}}={\theta }_{{{{{{\rm{SH}}}}}}}/{t}_{{{{{{\rm{s}}}}}}}=1.06\,{{{{{{\rm{nm}}}}}}}^{-1}$, where ${{J}_{{{{{{\rm{e}}}}}}}}^{{{{{{\rm{2D}}}}}}}$ represents the 2D electric current density, and *t*_s_ represents the surface thickness of TI [1.5 nm for (BiSb)_2_Te_3_]^31,44^.

### SOT-induced ferromagnetic resonance

**Fig. 4:**
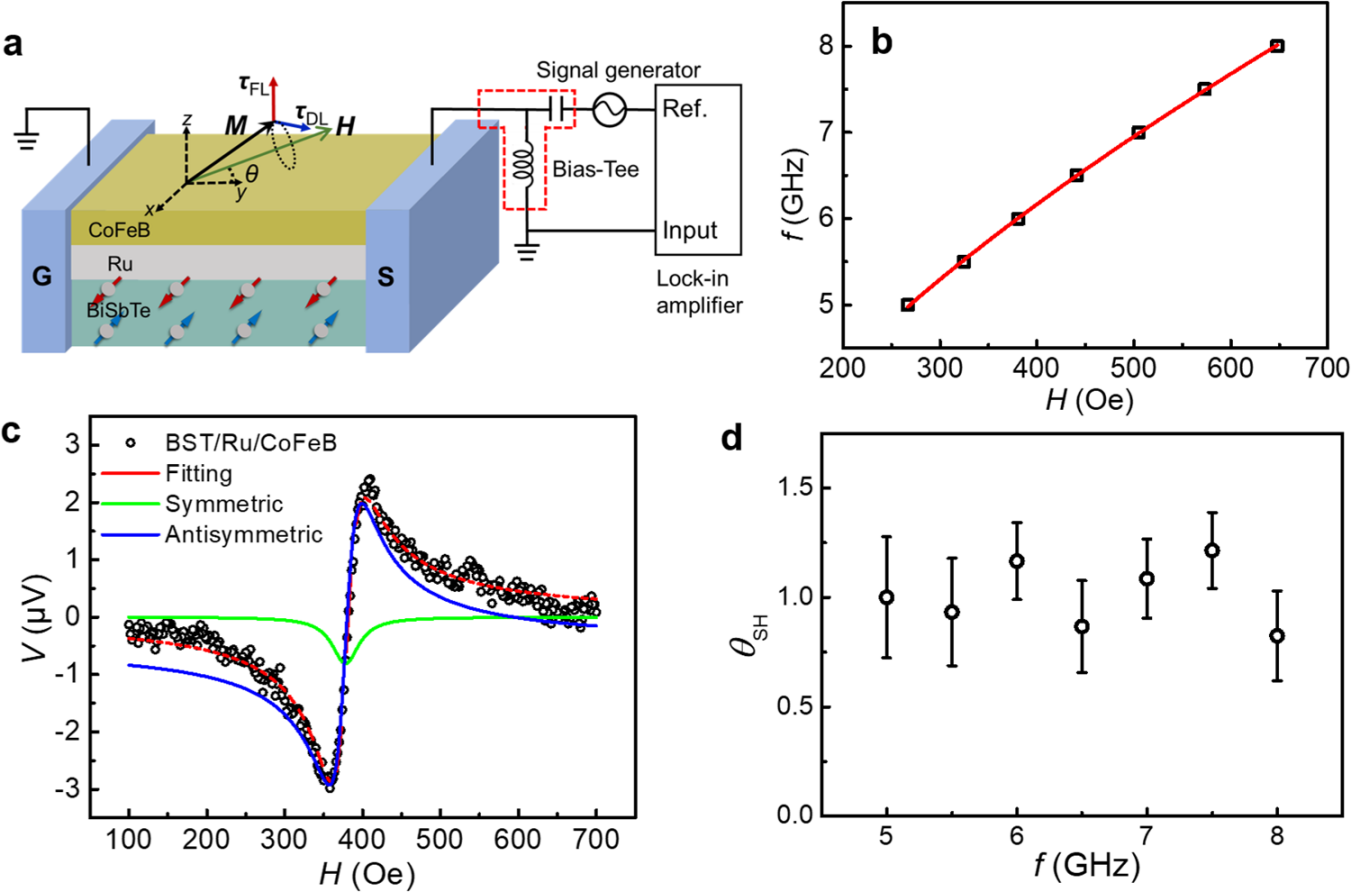
SOT-induced ferromagnetic resonance (FMR). **a** Schematic of the SOT-induced FMR measurement. A microwave current is applied to excite the magnetic resonance, and an applied in-plane magnetic field *H* with *θ* = 45° is scanned during the measurement. The damping-like torque *τ*_DL_ is dominated by the SOT, where the field-like torque *τ*_FL_ mainly comes from the Oersted field. **b** Resonance frequency *f* as a function of the magnetic field *H*, which can be fitted by the Kittel equation. **c** The *V*_mix_-*H* curve can be fitted by Eq. (1), where the SOT and Oersted field contributions can be obtained by the symmetric and antisymmetric parts, respectively. **d** The charge-spin conversion efficiency *θ*_SH_ is almost independent with the frequency from 5 to 8 GHz. The error bar comes from the fitting process in Fig. 4c.

The SOT-induced ferromagnetic resonance (ST-FMR)^23,34,45^ is also employed to quantify the charge-spin conversion efficiency in (BiSb)_2_Te_3_, with the structure of (BiSb)_2_Te_3_(10)/Ru(5)/CoFeB(2.5)/MgO(1.9), which is the same as the free layer in the TI-MTJ stack. As shown in the schematic of the ST-FMR measurement in Fig. 4a, a microwave current (12 dBm) is applied to excite the magnetic resonance, where an in-plane magnetic field *H* is scanned with a fixed angle *θ* = 45°. The microwave is modulated by the lock-in amplifier with a 20 kHz frequency. The damping-like torque *τ*_DL_ and the field-like torque *τ*_FL_ originate from the SOT and the Oersted field of the ac current, respectively. Figure 4b shows the magnetic resonant frequency *f* as the function of the magnetic field *H*, which can be fitted well by the standard Kittel equation ${f}=\frac{\gamma} {2{{{{{\rm{\pi}}}}}}}\sqrt{H_{{{{{\rm{res}}}}}} ({H}_{{{{{\rm{res}}}}}} +4{{{{{{\rm{\pi}}}}}}}{M}_{{{{{{\rm{eff}}}}}}})}$ with an effective in-plane magnetization 4π*M*_eff_ ~ 1.19 T.

During the magnetization precession, the mixing of the oscillation magnetoresistance and the ac current produces a dc voltage *V*_mix_, as shown in the Fig. 4c, which can be fitted by ${V}_{{{{{\rm{mix}}}}}}=S\frac{{\triangle}^{2}}{{\triangle}^{2}{+}{({H}_{{{{{\rm{ext}}}}}}-{H}_{{{{{\rm{res}}}}}})}^{2}}{+}A\frac{\triangle ({H}_{{{{{\rm{ext}}}}}}-{H}_{{{{{\rm{res}}}}}})}{{\triangle }^{2}{+}{({H}_{{{{{\rm{ext}}}}}}-{H}_{{{{{\rm{res}}}}}})}^{2}}$, where Δ is the linewidth, *H*_res_ is the resonant magnetic field, *S* and *A* represents the coefficient of symmetric part and antisymmetric part, respectively. The symmetric part *S* is attributed to the SOT (*τ*_DL_) from (BiSb)_2_Te_3_, which is proportional to the current density *J*_TI_; on the other hand, the antisymmetric part *A* comes from the Oersted field contribution (*τ*_FL_), which is dominated by the current density *J*_Ru_ in the Ru layer. The charge-spin conversion efficiency *θ*_SH_ could be expressed as:

1 $${\theta }_{{{{{{\rm{SH}}}}}}}=\frac{{J}_{{{{{{\rm{Ru}}}}}}}}{{J}_{{{{{{\rm{TI}}}}}}}}\frac{S}{A}\frac{e{\mu }_{0}{M}_{s}{t}_{{{{{{\rm{Ru}}}}}}}{t}_{{{{{{\rm{CoFeB}}}}}}}}{\hbar }\sqrt{1+(4{{{{{\rm{\pi }}}}}}{M}_{{{{{{\rm{eff}}}}}}}/{H}_{{{{{{\rm{res}}}}}}})}$$

where *t*_Ru_ and *t*_CoFeB_ represent the thickness of Ru and CoFeB, respectively. After subtracting the spin pumping contribution (27% of the symmetric part, Supplementary Note 6), the obtained *θ*_SH_ by ST-FMR for *f* = 5-8 GHz is shown in the Fig. 4d, and the *θ*_SH_ is almost constant at different frequencies, with the average value of 1.02 (*q*_ICS_ = 0.68 nm^−1^), which is consistent with *θ*_SH_ = 1.59 (*q*_ICS_ = 1.06 nm^−1^) from the SOT-induced switching field shift in the TI-MTJ device.

### All sputtered BiSb-MTJ device

**Fig. 5:**
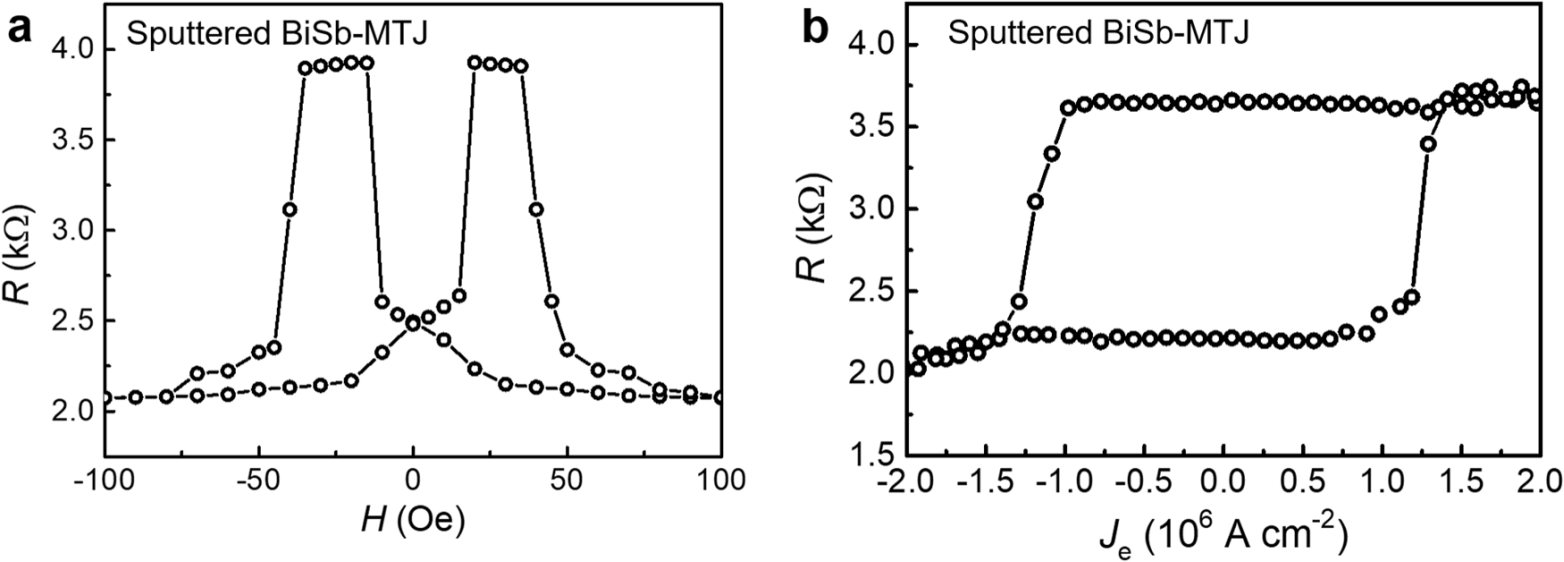
SOT switching in all sputtered BiSb-MTJ. **a** *R*-*H* curve for the all sputtered BiSb-MTJ device. **b** Current-driven SOT switching in the all sputtered BiSb-MTJ device (1 µm × 3 µm MTJ size, and ***σ***∥EA).

In order to be compatible with the industry-level manufacture, the topological insulator of BiSb is prepared by the magnetron sputtering method^29,46^, and the MTJ stack of Ru(5)/CoFeB(2.5)/MgO(2)/CoFeB(5)/Ta(8)/Ru(7) is in-situ deposited on top of the sputtered BiSb(10) without breaking the vacuum (thickness in nanometers), i.e., all sputtered BiSb-MTJ. The *TMR* ratio of the all sputtered BiSb-MTJ is around 90%, as shown in Fig. 5a. The current-induced SOT can efficiently switch the bottom CoFeB layer and thus the resistance state of MTJ at room temperature, with a critical switching current density *J*_c_ of 1.4 × 10^6^ A cm^−2^, as shown in Fig. 5b. Even *J*_c_ for the sputtered BiSb is 1 order of magnitude higher than the MBE-grown (BiSb)_2_Te_3_, the high conductivity of the sputtered BiSb (1.8 × 10^5^ Ω^−1^ m^−1^) could help to reduce the Ohmic loss, especially from the shunting effect.

In conclusion, the SOT-MRAM cell is demonstrated in a TI-MTJ device with over 100% *TMR* ratio, where the energy consumption is significantly reduced due to the ultralow switching current density of 10^5^ A cm^−2^. The >1 charge-spin conversion efficiency (*θ*_SH_) in (BiSb)_2_Te_3_ is quantified by the SOT-induced switching field shift and the ST-FMR measurements at room temperature, which breaks down the limitation of *θ*_SH_ < 1 in classical material systems. This work paves a path for the application of TI-driven magnetic memory, and potentially inspires the revolution of current magnetic memory technology from classical to quantum materials.

## Methods

### Sample growth and device fabrication

The high-quality (Bi_0.2_Sb_0.8_)_2_Te_3_ films were grown on Al_2_O_3_(0001) substrates by using a Perkin Elmer MBE system in ultrahigh vacuum. Before the growth, the substrate was pre-annealed in the vacuum chamber at up to 700 °C to clean the surface. High-purity Bi (99.9999%), Te (99.9999%) and Sb (99.999%) were co-evaporated by conventional effusion cells and cracker cells. During the TI growth, the substrate was maintained at 200 °C, where the Bi, Sb and Te cells were kept at 457 °C, 387 °C and 340 °C, respectively. The layer-by-layer epitaxial growth was monitored by the in-situ reflection high-energy electron diffraction (RHEED).

The MTJ stacks of Ru (5 nm)/CoFeB (2.5 nm)/MgO (1.9 nm)/CoFeB (5 nm)/Ta (8 nm)/Ru (7 nm) were deposited by using a Singulus ROTARIS magnetron sputtering system at room temperature with a base pressure of 1 × 10^−6^ Pa. The CoFeB denotes Co_40_Fe_40_B_20_ alloy with nominal target compositions. When depositing the CoFeB ferromagnetic layers, a magnetic field of 50 Oe was applied to induce the magnetic easy axis. The MTJ devices with a rectangular shape were fabricated by using two photolithography, one electron-beam lithography and two Ar ion milling steps. To avoid the chemical degradation of the TI films caused by the developer, a poly(methyl methacrylate) (PMMA) layer with a thickness of 300 nm was spin-coated on top of the film before the photolithography step, and then removed by O_2_ plasma before the Ar ion milling step. Subsequently, the final patterned MTJ devices were annealed in the vacuum at 300 °C for 1 h with a magnetic field of 8 kOe.

### Magnetic and spin transport measurements

The SOT switching in the 3-terminal TI-MTJ device was measured by the probe station system with an electromagnet, where the pulse current was applied by the Keithley 2612 current source, and the voltage through the MTJ was measured by a Keithley 2182 A voltmeter. For the ST-FMR measurement, a signal generator was used to apply the microwave current with a nominal power of 12 dBm, where a lock-in amplifier (Stanford Research SR-830) was used to measure the voltage. In order to improve the signal-to-noise ratio, the microwave current was modulated by a sine function from the lock-in amplifier with 20 kHz. The magnetic properties of the TI-MTJ stack were measured by a vibrating sample magnetometer (VSM) system. All measurements were performed at room temperature.

## Supplementary information

Supplementary Information

## Data Availability

The data that support the findings of this study are available from the corresponding authors upon reasonable request.
