# Supplementary material for: Magnetic memory driven by topological insulators
Source: Nat Commun. 2021 Oct 29;12:6251. doi: 10.1038/s41467-021-26478-3 (PMC8556271; doi:10.1038/s41467-021-26478-3)
Supplement: Supplementary file 1 — Supplementary Information [file 41467_2021_26478_MOESM1_ESM.pdf]

Supplementary Information

Magnetic memory driven by topological insulators

Hao Wu[1*†], Aitian Chen[2†], Peng Zhang[1†], Haoran He[1], John Nance[3], Chenyang Guo[4], Julian Sasaki[5,6], Takanori Shirokura[5,6], Pham Nam Hai[5,6], Bin Fang[2], Seyed Armin Razavi[1], Kin Wong[1], Yan Wen[2], Yinchang Ma[2], Guoqiang Yu[4], Gregory P. Carman[3], Xiufeng Han[4], Xixiang Zhang[2], and Kang L. Wang[1*]

[1]*Department of Electrical and Computer Engineering, and Department of Physics and Astronomy, University of California, Los Angeles, California 90095, United States*

[2]*Physical Science and Engineering Division, King Abdullah University of Science and Technology, Thuwal 23955-6900, Saudi Arabia*

[3]*Department of Mechanical and Aerospace Engineering, University of California, Los Angeles, California 90095, USA*

[4]*Beijing National Laboratory for Condensed Matter Physics, Institute of Physics, Chinese Academy of Sciences, Beijing 100190, China*

[5]*Department of Electrical and Electronic Engineering, Tokyo Institute of Technology, Tokyo 152-8550, Japan*

[6]*Center for Spintronics Research Network (CSRN), The University of Tokyo, Tokyo 113-8656, Japan*

*Corresponding author. E-mail: wuhaophysics@ucla.edu; wang@ee.ucla.edu

[†]These authors contributed equally to this work.

Supplementary Note 1: Micromagnetic simulations

A micromagnetic finite difference model is used to study the SOT-induced magnetization switching for the TI-MTJ device[1, 2], where the micromagnetic model can be represented by the Landau Lifshitz Gilbert (LLG) equation[3]:

$$\frac{1}{\gamma}\frac{\partial \boldsymbol{m}}{\partial t} = -\boldsymbol{m}\times\boldsymbol{H}_{\text{eff}} + \frac{\alpha}{\gamma}\left(\boldsymbol{m}\times\frac{\partial \boldsymbol{m}}{\partial t}\right) - \frac{J_s\hbar}{2eM_s t}\left(\boldsymbol{m}\times\left(\boldsymbol{m}\times\boldsymbol{\sigma}\right)\right) \tag{1}$$

Where $\boldsymbol{m}$ is the normalized magnetization vector, γ is the gyromagnetic ratio, α is the Gilbert damping factor, $\hbar$ is the reduced Planck constant, e is the elementary charge, and J_s is the spin current density. $\boldsymbol{\sigma}$ is the spin polarization, t is the thickness of the magnetic layer, and M_s is the saturation magnetization. Here, a spin Hall angle θ_{SH} of 2.0 is assumed. $\boldsymbol{H}_{\text{eff}}$ is the effective field and consists of three components: $\boldsymbol{H}_{\text{eff}} = \boldsymbol{H}_{\text{ex}} + \boldsymbol{H}_{\text{d}} + \boldsymbol{H}_{\text{k}}$, where $\boldsymbol{H}_{\text{ex}}$ is the exchange field, $\boldsymbol{H}_{\text{d}}$ the demagnetization field, and $\boldsymbol{H}_{\text{k}}$ the magnetic anisotropy field.

The easy axis (EA) of the MTJ device is set along the y direction for the collinear $\boldsymbol{\sigma}$ // EA case and the x direction for the orthogonal $\boldsymbol{\sigma}\perp$EA case, respectively, as shown in Supplementary Fig. 1a and 1d. Supplementary Figure 1a-c and 1d-f show the results of the $\boldsymbol{\sigma}$ // EA and $\boldsymbol{\sigma}\perp$EA cases, respectively. In Supplementary Fig. 1b, the magnetic moment firstly forms an oscillation state, and then precessionally switches from $+m_y$ to $-m_y$ at about 7.5 ns, i.e., precessional switching mode. Supplementary Figure 1c shows the field-free SOT switching, where the damping-like torque [$-\boldsymbol{m}\times\left(\boldsymbol{m}\times\boldsymbol{\sigma}\right)$] can break the inversion symmetry between $+m_y$ and $-m_y$ for the collinear $\boldsymbol{\sigma}$ and EA. While in the $\boldsymbol{\sigma}\perp$EA case, the magnetic moment dynamically switches from $+x$ to the $\boldsymbol{\sigma}$ ($+y$) direction firstly, and then switches to the EA ($-x$) direction due to the inversion symmetry

breaking by an out-of-plane magnetic field H_z, i.e., dynamic reversal mode, as shown in Supplementary Fig. 1e, which can be indicated by the opposite SOT switching polarities at $H_z = \pm 100$ Oe in Supplementary Fig. 1f. The dynamic reversal mode in the $\boldsymbol{\sigma} \perp$ EA case produces a much shorter switching trajectory and a much faster switching speed (1.0 ns), while the critical switching current density J_c is much higher, as shown in Supplementary Fig. 1e and 1f.

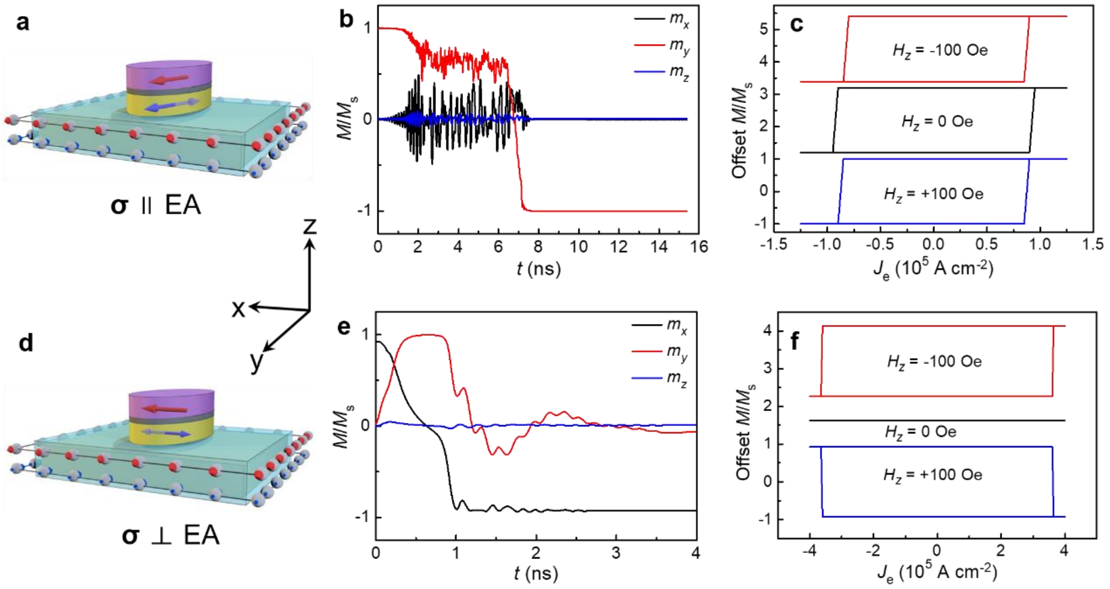

Supplementary Fig. 1. Micromagnetic simulation results. a-c show the results of the collinear $\boldsymbol{\sigma}$ // EA case. b shows the precessional switching process of the SOT-induced magnetization switching. c shows the magnetization as a function of the current density J_e, where a series of H_z is applied during the switching. d-f shows the results of the orthogonal $\boldsymbol{\sigma} \perp$ EA case. e and f show the M/M_s-t and M/M_s-J_e curves, respectively.

Supplementary Note 2: HAADF and EDS mapping of the TI-MTJ stack

Supplementary Fig. 2 shows the high-angle annular dark field (HAADF) and the energy dispersive spectroscopy (EDS) mapping of the

(BiSb)$_2$Te$_3$(10)/Ru(5)/CoFeB(2.5)/MgO(1.9)/CoFeB(5)/Ta(8)/Ru(7) (thickness in nanometers) multilayer stack, which demonstrate the layer-by-layer structure of (BiSb)$_2$Te$_3$ and the clear interface between the (BiSb)$_2$Te$_3$ and the MTJ stack.

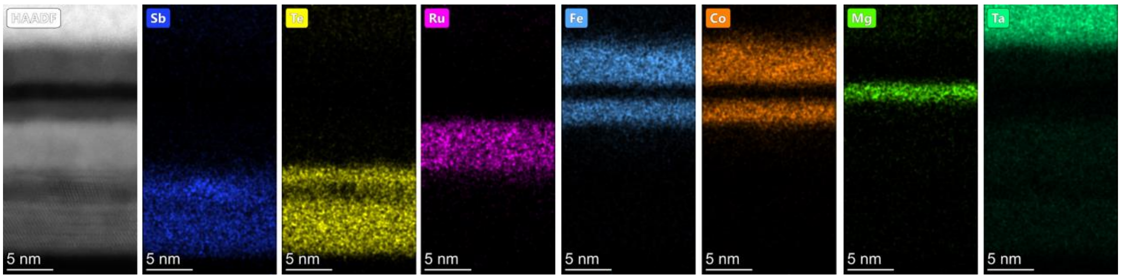

Supplementary Fig. 2. HAADF and EDS mapping of the TI-MTJ multilayer stack.

Supplementary Note 3: Transport characterization of (BiSb)$_2$Te$_3$

The temperature dependence of the sheet resistance $R_s = \rho_{xx} / t$ of (BiSb)$_2$Te$_3$ in Supplementary Fig. 3a shows the semiconducting properties, and the surface states dominate at lower temperature. From the Hall signals in Supplementary Fig. 3b, we can obtain the 2-dimeantional (2-D) carrier density of 2.1×10^{12} cm^{-2} at 5 K and 8.6×10^{12} cm^{-2} at 300 K, respectively.

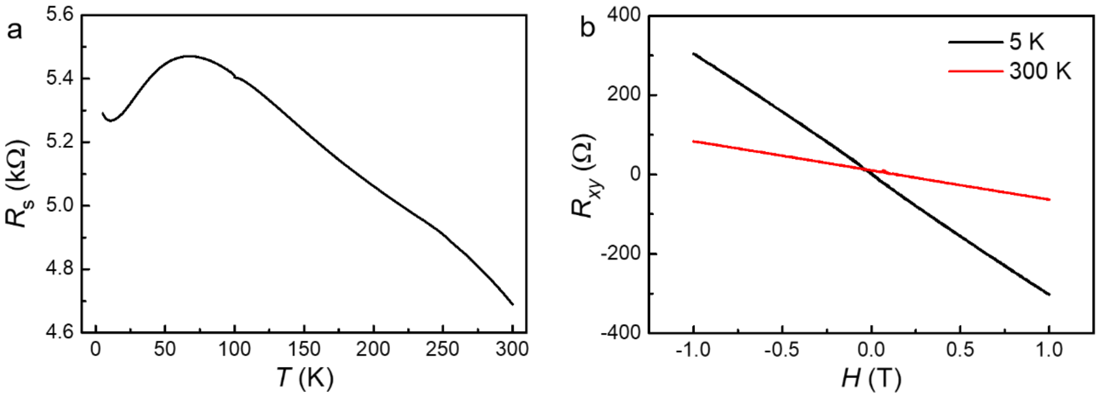

Supplementary Fig. 3. a, Temperature dependence of the sheet resistance R_s in (BiSb)$_2$Te$_3$. b, The Hall resistance R_{xy} as a function of the magnetic field H at 5 K and 300 K, respectively.

Supplementary Note 4: Possible SOT contribution from Ru

We perform the current-driven SOT switching measurement in the MTJ device (Ru/CoFeB/MgO/CoFeB/Ta/Ru) without the topological insulator of $(BiSb)_2Te_3$, as shown in Supplementary Fig. 4a. In this case, there is no switching even the current density reaches 4×10^7 A cm^{-2}, indicating the negligible SOT contribution from Ru[4]. Also, the SOT-induced ferromagnetic resonance (ST-FMR) is measured in the Ru/CoFeB/MgO stack (Supplementary Fig. 4b) without TIs, where the tiny symmetric/antisymmetric contribution also indicates that the SOT from Ru is negligible.

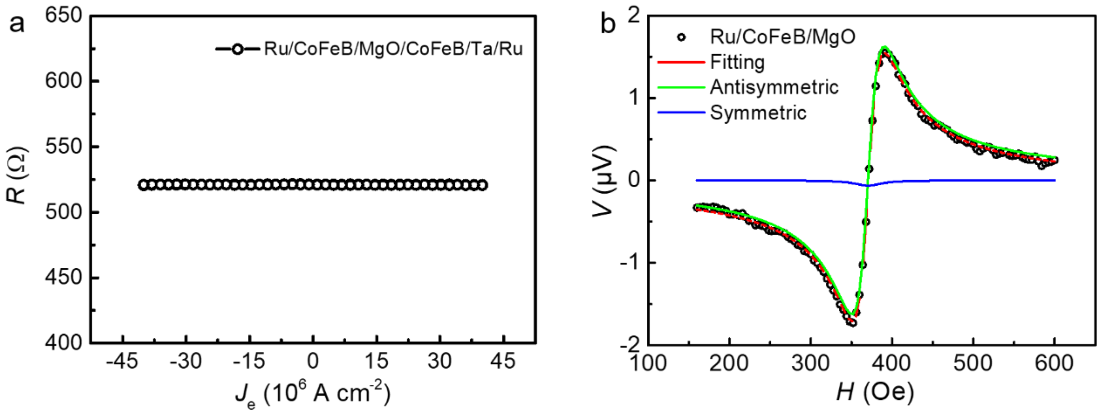

Supplementary Fig. 4. a, The current-driven SOT switching result for a MTJ device (Ru/CoFeB/MgO/CoFeB/Ta/Ru) without TIs. b, ST-FMR results for the Ru/CoFeB/MgO stack without TIs.

Supplementary Note 5: Ru thickness dependence of θ_{SH}

The Ru thickness between $(BiSb)_2Te_3$ and CoFeB is changed from 3 nm to 9 nm, and the ST-FMR results are shown in Supplementary Fig. 5. The charge-spin conversion efficiency θ_{SH} shows a maximum at the sample with Ru = 5 nm, and then decreases with increasing the Ru thickness because of the spin current loss. The θ_{SH} of the sample with Ru = 5 nm is slightly enhanced compared to that with Ru = 3 nm, which may come

from the decoupling of exchange interaction between CoFeB and topological surface states that damages the spin-momentum locking.

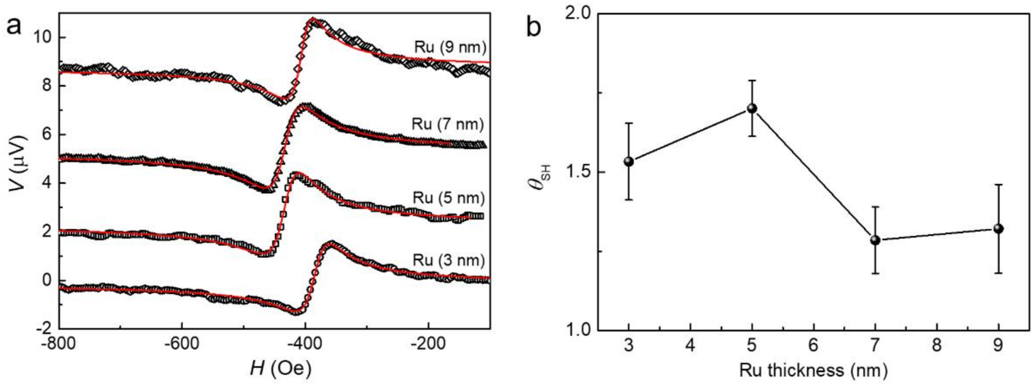

Supplementary Fig. 5. a, ST-FMR for $(BiSb)_2Te_3$/Ru(t)/CoFeB/MgO samples with the thickness t of Ru from 3 nm to 9 nm. b, Charge-spin conversion efficiency θ_{SH} as a function of Ru thickness.

Supplementary Note 6: Spin pumping contribution in the ST-FMR measurement

To estimate the contribution of signal from spin pumping in the ST-FMR measurement, i.e., inverse spin Hall effect, the magnetic field-driven FMR experiment is performed in a similar device, where an insulating layer is used to electrically separate the waveguide and the magnetic device. The signal is dominated by symmetric part, as shown in Supplementary Fig. 6, and the spin pumping contribution is estimated around 0.27 μV, which is around 27% of the symmetrical part of the ST-FMR signal. Based on this, we have also considered the spin pumping contribution in the ST-FMR data and modify the obtained spin Hall angle of TIs in the manuscript and supplementary information.

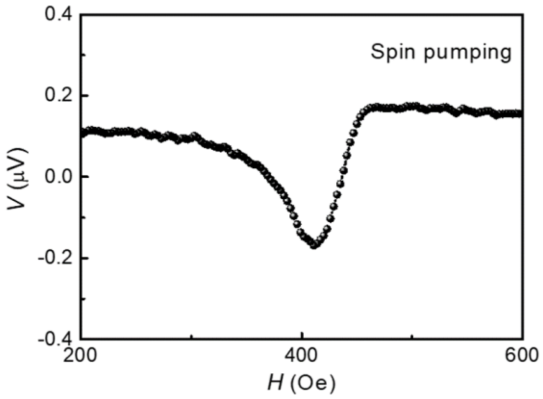

Supplementary Fig. 6. Spin pumping signals in BST/Ru/CoFeB/MgO/Ta stacks at 6.5 GHz, with the magnetic field-driven FMR.

Supplementary Note 7: Pulse width dependence of SOT switching

We perform the SOT switching of the TI-MTJ device with varied pulse widths of writing current from 10 ms to 10 ns, as shown in Supplementary Fig. 7a and 7b. The switching current density J_c is gradually increased at a much shorter pulse width (around 2 times from 10 ms to 10 ns), which is a typical feature of the thermal activation range.

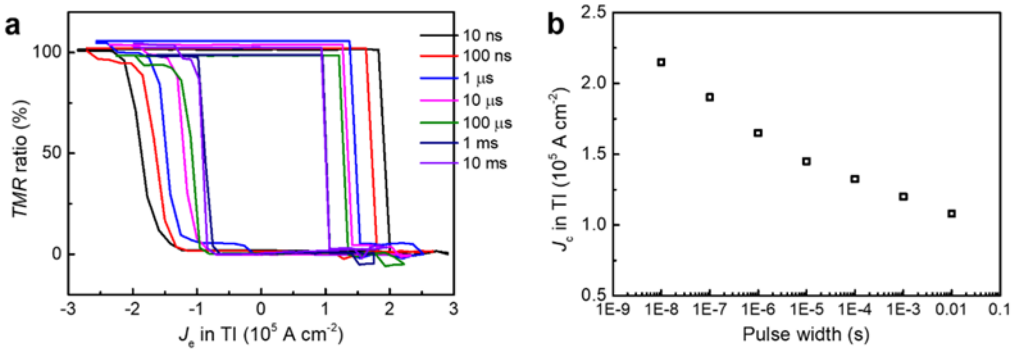

Supplementary Fig. 7. a, SOT switching with varied pulse widths of writing current from 10 ns to 10 ms. b, Switching current density as a function of the pulse width.

Supplementary Note 8: Thermal stability test of TI-MTJ device

In order to measure the thermal stability factor Δ in TI-MTJ device, we perform the switching probability P as a function of the applied magnetic field H, by using the

relation based on Stoner-Wohlfarth model[5]:

$$P(\tau) = 1 - exp\left[-\frac{\tau}{\tau_0} exp\left\{-\Delta\left(1 - \frac{H-H_s}{H_k^{eff}}\right)^2\right\}\right],$$

Where τ is the pulse duration time (1 s) of H, τ_0 is the inverse of attempt frequency (1 ns), H_k^{eff} is the effective anisotropy field.

For experiment, the minor loops of *TMR-H* are measured for 50 times, as shown in Supplementary Fig. 8a, where only the free layer is switched; and thus, to obtain the switching probability P as a function of the magnetic field H, as shown in Supplementary Fig. 8b. After fitting the *P-H* curve with above equation, we can obtain a thermal stability factor $\Delta = 61$.

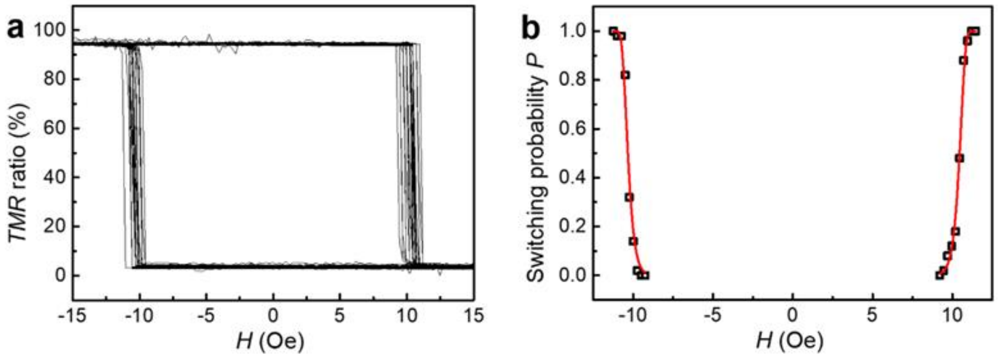

Supplementary Fig. 8. a, Minor *TMR-H* loops measured for 50 times. b, Switching probability P of the free layer as a function of the applied magnetic field H.

Supplementary Note 9: Current distribution estimation

The following Supplementary Table 1 shows the resistivity, thickness, and writing current distribution in different layers of the SOT channel for TI-MTJ device.

Supplementary Table 1:

	$(BiSb)_2Te_3$	Ru	CoFeB
Resistivity (μΩ cm)	4680	25	165

Thickness	10	5	2.5
Percentage of current	1%	92%	7%

Supplementary Note 10: Bulk and surface states contributions

Supplementary Figure 9 shows the thickness dependent transport data of $(BiSb)_2Te_3$ (BST) with the same growth recipe. With BST thickness t increasing from 4 nm to 12 nm, the sheet resistance R_s decreases monotonically, indicating the increasing portion of bulk conduction involvement, as shown in Supplementary Fig. 9a. For the thickness dependence (t) of resistivity (ρ) in Supplementary Fig. 9b, it shows a similar trend with Fig. 1d in the previous report[6], i.e., increases and then saturates, which indicates the surface is more conducting than bulk.

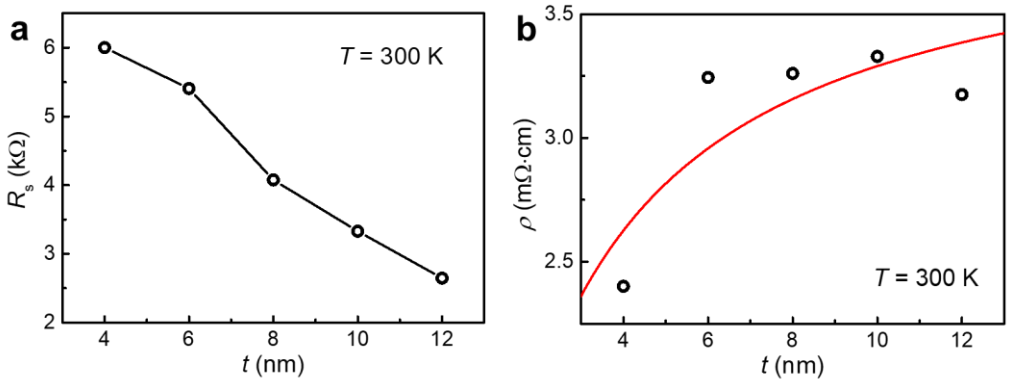

Supplementary Fig. 9. (a) Sheet resistance R_s and (b) resistivity ρ for BST thickness from 4 nm to 12 nm measured at 300 K.

We can estimate the surface and bulk contributions for the electron conduction: $\frac{1}{\rho}=\frac{1}{\rho_s}\cdot\frac{2t_s}{t}+\frac{1}{\rho_b}\cdot\frac{(t-2t_s)}{t}$, where the 1st and 2nd terms represent the surface and bulk conductions (surface thickness t_s =1.5 nm for BST), respectively. After fitting the ρ-t curve with the above equation, we can get the surface resistivity (2.36 mΩ cm) and bulk resistivity (3.96 mΩ cm), and therefore, the surface and bulk contributions for 6 nm and

10 nm BST we used in our SOT devices can be obtained (300 K), as shown in the following Supplementary Table 2. It should be noted that the bulk band gap of BST is small ~ 0.2 eV, so there would still be a considerable portion of bulk states contribution at room temperature (300 K).

Supplementary Table 2:

BST thickness	6 nm	10 nm
Surface states contribution (300 K)	63%	42%
Bulk states contribution (300 K)	37%	58%

References:

1. Vansteenkiste, A. et al. The design and verification of MuMax3. *AIP Advances* **4**, 107133 (2014).
2. Wang, Q. et al. Strain-Mediated Spin-Orbit-Torque Switching for Magnetic Memory. *Physical Review Applied* **10**, 034052 (2018).
3. Gilbert, T.L. A phenomenological theory of damping in ferromagnetic materials. *IEEE Transactions on Magnetics* **40**, 3443-3449 (2004).
4. Wen, Z., Kim, J., Sukegawa, H., Hayashi, M. & Mitani, S. Spin-orbit torque in Cr/CoFeAl/MgO and Ru/CoFeAl/MgO epitaxial magnetic heterostructures. *AIP Advances* **6**, 056307 (2016).
5. Sato, H. et al. CoFeB Thickness Dependence of Thermal Stability Factor in CoFeB/MgO Perpendicular Magnetic Tunnel Junctions. *IEEE Magnetics Letters* **3**, 3000204-3000204 (2012).
6. Barua, S. & Rajeev, K.P. Status of surface conduction in topological insulators. *AIP Advances* **4**, 017135 (2014).
